# Evidence synthesis evaluating body weight gain among people treating HIV with antiretroviral therapy - a systematic literature review and network meta-analysis

**DOI:** 10.1016/j.eclinm.2022.101412

**Published:** 2022-05-12

**Authors:** Steve Kanters, Francoise Renaud, Ajay Rangaraj, Kenneth Zhang, Eve Limbrick-Oldfield, Monica Hughes, Nathan Ford, Marco Vitoria

**Affiliations:** a School of Population and Public Health, University of British Columbia, 2206 E Mall, Vancouver, British Columbia, Canada; b RainCity Analytics, Vancouver, Canada; c Global HIV, Hepatitis and Sexually Transmitted Infections Programmes, World Health Organization, Geneva, Switzerland; d School of Health and Related Research, University of Sheffield, Sheffield, United Kingdom

**Keywords:** HIV, Body weight gain, Dolutegravir, Tenofovir alafenamide, Systematic review, Network meta-analysis, Prognostic factors

## Abstract

**Background:**

This systematic review aimed to compare body weight gain associated outcomes over time between dolutegravir (DTG)-based antiretroviral (ART) regimens to other ART regimens, to compare tenofovir alafenamide (TAF)-based regimens, and to evaluate the associated prognostic factors.

**Methods:**

Systematic searches of MEDLINE, Embase, and CENTRAL for RCTs and observational studies comparing ART regimens were conducted on 13 September 2021. Outcomes of interest included: change in body weight, body mass index (BMI), waist circumference; and risk of hyperglycaemia and diabetes. Network meta-analyses were conducted at 12, 24, 48, 96 and 144 weeks using two networks differentiated by 3rd agents and backbone agents.

**Findings:**

The review identified 113 publications reporting on 73 studies. DTG-based regimens led to statistically higher weight gains than efavirenz-based regimens at all time points (mean difference: 1·99 kg at 96 weeks; 95% credible interval: 0·85–3·09) and was higher over time than low-dose efavirenz-, elvitegravir-, and rilpivirine-based regimens. They were comparable to raltegravir-, bictegravir- and atazanavir-based regimens. For backbones, TAF led to higher weight gain relative to tenofovir disoproxil fumarate (TDF), abacavir, and zidovudine. Prognostic factor analysis showed both low CD4 cell count and high HIV RNA viral load at baseline were consistently associated with higher weight gain, while sex was an effect modifier to African origins.

**Interpretation:**

DTG-based regimens lead to larger average weight gains than some other ART regimens and TAF leads to larger average weight gains than all other backbone antiretrovirals. Further research is needed to better understand long-term outcomes and their relationship to other metabolic outcomes.

**Funding:**

The WHO Global HIV, Hepatitis and Sexually Transmitted Infections Programmes.

**Research in context**

**Evidence before this study**

In 2019, in reviewing the evidence to support the recommendation to move to DTG-based regimens for preferred 1st and 2nd line treatments in the updated WHO consolidated HIV guidelines, the ADVANCE trial provided evidence of potentially higher weight gain in patients using DTG- and/or TAF-based regimens. With this growing concern, more and more studies have explored weight gains among people living with HIV on ART. Prior to conducting our study, we conducted a search of PubMed for studies published through to 10 September 2021, using the search terms “HIV”, “Weight” and “meta-analysis” confirmed no all-encompassing meta-analyses and suggested that there remains a need for further clarity on the topic.

**Added value of this study**

Our comprehensive meta-analysis establishes a moderate certainty of larger weight gains using DTG-based regimens relative to efavirenz, other NNRTIs and select other regimens, while being comparable to others including most integrase inhibitors. The study also found moderate to high certainty that TAF leads to larger weight gains than all other NRTIs combined to lamivudine/emtricitabine. Finally, by reviewing multiple prognostic factor analyses side-by-side, it showed that low CD4 cell counts and high HIV RNA are the most consistent prognostic factors, while the interaction between sex and African origins lead to less consistent results.

**Implications of all the available evidence**

Increased body weight gain continue to be a concern for DTG, TAF and the other antiretrovirals that are comparable to them. As research moves forward, better understanding the prognostic factors of weight gains outside of healthy ranges (i.e., excluding return to health and other reasonable weight gains) will be important to better contextualize these weight gains and their concerns to existing policies and guidance.

Alt-text: Unlabelled box

## Introduction

HIV/AIDS is one of the most studied and best characterized pandmeics,^1^ with the highest death toll in human history.^2^ One of the key advancements in the fight against HIV was the development of antiretroviral therapy (ART), rendering HIV into a chronic disease in the last decade.^3^ A unique feature of this pandemic is that it requires life-long therapy for those afflicted by it. With respect to HIV treatment, there have been many improvements over the years; from the reduced pill burden to the accessibility of today's test and treat approach.^4^ One important recent change to HIV treatment has been the shift from efavirenz (EFV)-based ART regimens to dolutegravir (DTG)-based ART regimens as the preferred approach.^5^^,^^6^

This shift in treatment patterns was motivated by the higher efficacy and tolerability of DTG-based ARTs, and was accelerated by both the important reduction in generic formulation costs and the change in clinical guidelines.^6^^,^^7^ Prior to the 2019 WHO recommendation,^8^ preliminary data from Botswana (Tsepamo study) raised the concern over the possible correlation between DTG use by women at the time of conception and occurrence of neural tube defects in their infants.^9^ Recent additional data from the Tsepamo study demonstrated that the prevalence of NTDs with preconception DTG no longer significantly different than observed with non-DTG preconception exposures.^9^, ^10^, ^11^, ^12^ However, two trials looking at benefits and risk in large adult populations showed evidence of weight gain among DTG users.^13^ The ADVANCE trial also suggested greater weight gain among patients using a tenofovir alafenamide (TAF) based backbone relative to one that is tenofovir disoproxil fumarate (TDF)-based.

Body weight gain in people living with HIV (PLWH) is a complex, multifactorial issue. Risks of obesity and overweight in PLWH have been documented globally.^14^, ^15^, ^16^, ^17^ Excess weight gain is a multifactorial process, caused by the interactions between the modern obesogenic environment, HIV metabolic immune response, ART regimen composition, and demographic factors.^18^^,^^19^ It is known that certain ART regimens use has led to weight gain in PLWH, but it is unclear whether these weight gains are due to an improvement in health or an unintended side effect from ART use.^20^^,^^21^ Another unknown is if weight gains plateau or are sustained after ART.^22^ Until recently, there has been very little research conducted on the effects of ART regimens on body weight. Now, current evidence points towards DTG, other integrase inhibitors, and TAF being treatments with a higher risk of weight gain.^19^^,^^23^, ^24^, ^25^ This recent rise of publications on ART and weight gain warranted the need for a systematic literature review (SLR) and meta-analysis.

Understanding the mechanisms of body weight gain in PLWH is critical. Excessive weight gain caused by ART regimens could create greater cardiovascular disease risk and metabolic comorbidities.^22^^,^^26^ Rates of diabetes mellitus have been observed to be higher in PLWH, especially those who gained weight after ART inititation.^27^, ^28^, ^29^, ^30^ An assessment of weight gain due to ART regimens is needed to ensure the optimal treatment is delivered to PLHIV.

We sought to evaluate how different ART regimens compare with respect to body weight gains over time. This review evaluates how DTG-based regimens compare to other regimens and how TAF-based backbones compare to others. Additionally, we explored the risk factors associated with weight gains.

## Methods

This study sought to identify the comparative evidence of weight gain and related outcomes between different ART regimens, with a focus on DTG and TAF. We used an SLR to identify the evidence and utilized the same search to identify prognostic-factor analyses for weight gain among PLWH treated with ART.

### Systematic literature review

On 13th September 2021, we conducted systematic searches in MEDLINE, EMBASE, and CENTRAL using Ovid. Searches involved terms pertaining to the study design, population, interventions, and outcomes. The following conferences were also searched: the 2020 and 2021 Conferences on Retroviruses and Opportunistic Infections (CROI); the 2021 AIDS conference; the 2020 International AIDS Society (IAS) conference; and the 2019–2021 HIV Glasgow conferences.

Eligible studies were randomized controlled trials (RCTs) and comparative observational studies that included adults and adolescents (aged 10 years or more) living with HIV initiating ART, regardless of prior experience or whether participants were virally suppressed. The outcomes of interest were body weight gain, change in body mass index (BMI), waist circumference change, change in visceral, fat hyperglycaemia, and diabetes mellitus. Eligible treatments were commonly used three- and two-agent ART regimens containing: EFV, low dose EFV (EFV_400_), DTG, raltegravir (RAL), cobicistat-boosted elvitegravir (EVG/c), bictegravir (BIC), doravirine (DOR), nevirapine (NVP), rilpivirine (RPV), or ritonavir-boosted atazanavir (ATV/r), ritonavir-boosted darunavir (DRV/r), and ritonavir-boosted lopinavir (LPV/r) for HIV treatment; each in combination with a NRTI backbone and each using the FDA approved dosage. For studies focusing on the comparison of NRTI backbone agents, eligible study-arms contained TAF, TDF, abacavir (ABC), or zidovudine (AZT). Older agents, such as stavudine were ineligible. Given that body weight gain is a more recent development, the search was restricted to 2011 to the present. Finally, were restricted to English language publications.

Both study selection and data extraction were done independently and in dual. The extracted data included study characteristics, patient characteristics, and outcomes. Quality assessment was conducted using the Cochrane Risk of Bias (Version 2) instrument and the Newcastle-Ottawa Scale (NOS) for randomized and observational studies, respectively.^31^^,^^32^ Further details regarding the specific search terms and steps of study selection and extraction are provided in the extended methods of the *Web Appendix*.

### Statistical analysis

When sufficient data were available, evidence was analysed using network meta-analyses (NMA). All NMAs were conducted using hierarchical Bayesian modelling. Fixed and random-effects models were used for all outcomes. The deviance information criterion (DIC) was used for model selection.^33^ Model fit was assessed using leverage plots and any outliers identified in this fashion were investigated further. Consistency was evaluated using edge-splitting^34^ and the difference in estimates generated by the two sets of evidence was evaluated with the Bucher test for inconsistency.^35^ The PRISMA NMA checklist was used to ensure reporting and analyses are compliant with best practices.

For modelling, we used logistic regression and linear regression models for binary and continuous outcomes, respectively. Body weight, BMI and waist circumference were analysed along each of their reported time points as continuous outcomes, while hyperglycaemia and diabetes outcomes were dichotomous. The logistic regression model used the logit link function and a binomial likelihood, with results presented as odds ratios (OR). Similarly, linear regression models used an identity link and normal likelihood, with results presented as mean differences. As additional summary measures, we estimated the treatment rankings using surface under the cumulative ranking curve (SUCRA) values.

**Figure 1:**
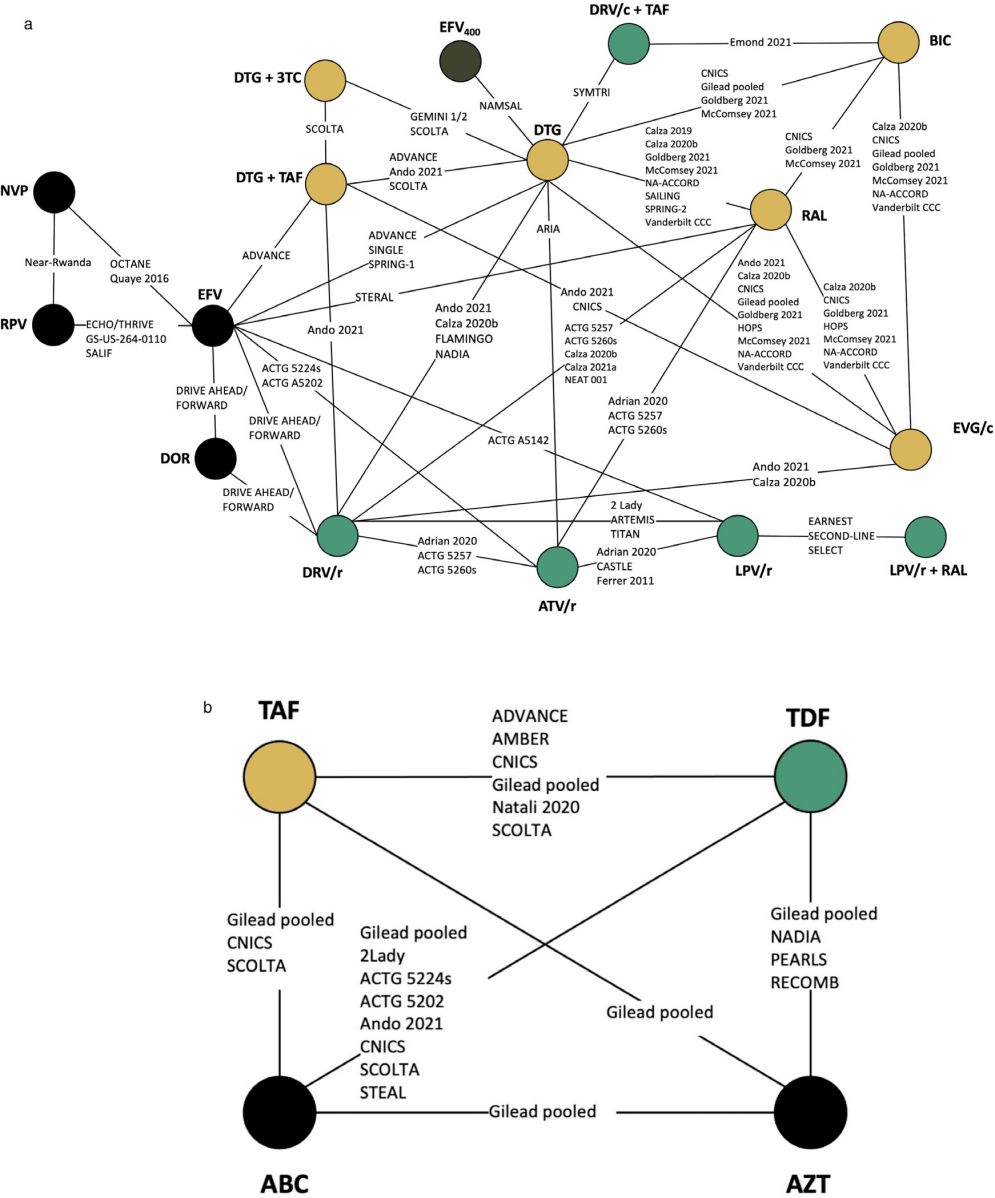
Evidence network of all studies included in the (A) primary network and (B) NRTI network. Each circle represents a treatment of interest. Each line represents the existence of comparative evidence between the two connected treatments. The names on each line represent studies providing the evidence. In network (A), the colours represent treatment classes: integrase inhibitors in gold, NNRTIs in black and PIs in green. BIC: bictegravir; BB: Back bone (mixed); DTG: dolutegravir; EFV: efavirenz; EVG/c: elvitegravir/cobicistat; RAL: raltegravir; DOR: Doravirine; DRV/r: Ritonavir-boosted darunavir; ATV/r: Ritonavir-boosted atazanavir; LPV/r: Ritonavir-boosted lopinavir; 3TC: Lamivudine; TAF: tenofovir alafenamide; TDF: Tenofovir disoproxil fumarate; ABC: Abacavir; AZT: Zidovudine.

Two networks were created based on the specific third agent combinations and non-XTC (lamivudine (3TC) or emtricitabine (FTC)) backbone. The primary network (Figure 1a) defined each node according to the third agent (integrase strand transfer inhibitor (INSTI), non-nucleoside reverse transcriptase inhibitor (NNRTI), or protease inhibitor (PI) and differentiated the backbone as being TAF/XTC, 3TC on its own, or another backbone (always two NRTIs). We considered having TDF/XTC as a backbone, but it led to a less well-connected network that reduced its utility. We were careful to include only comparisons with the same backbone when using the last category of backbone (e.g., EFV+TDF/XTC vs. DTG+ABC/XTC was not included as EFV vs. DTG). This approach assumes that the other agents do not interact with the NRTIs of interest.

In the backbone network (Figure 1b), each node was defined by a single agent: TAF, TDF, ABC, and AZT. Every comparison involved the very same additional agents. For example, EFV+TDF/XTC vs. EFV+TAF/XTC was simplified to TDF vs. TAF, just as DTG+TDF/XTC vs. DTG+TAF/XTC was. One key study for both networks was the pooled analysis of seven RCTs by Sax et al.^21^ These trials contain a variety of treatments and based on the reported analyses, they provide three sets of comparisons: DTG vs. BIC vs EVG/c, EFV vs RPV, and TAF vs. TDF vs. ABC vs. ZDV. As such, the data were analysed to reflect these sets of comparisons only.

Both regression adjustments and restricted analyses were used. We conducted meta-regression adjustments to evaluate whether differences in baseline CD4 cell count, baseline log transformed HIV viral load, proportion of females, proportion of people of African origin and baseline age led to improved model fit. Decisions over which models to choose were made on the basis of the DIC as described previously.^36^

Restricted analyses were used to address the mixed study designs used for these analyses (i.e., the inclusion of observational studies). The primary analyses included observational studies to make full use of the data, while sensitivity analyses used RCT evidence only. Hierarchical modelling that allows for down-weighting of the observational studies were not conducted.^37^ However, the case with and without the observational studies represent the two end cases with down-weighting necessarily leading to results that lie between these. Given the larger number of RCTs in the evidence base, this simpler approach was deemed sufficient.

We employed the Grading of Recommendations Assessment, Development and Evaluation (GRADE) system for rating the overall certainty of evidence.^38^, ^39^, ^40^, ^41^, ^42^, ^43^ The certainty of evidence for each main outcome was determined after considering direction and measure of effect, risk of bias, and sample size, and categorized as either high, moderate, low, or very low.^44^

In addition to our primary analysis, we also explored the prognostic factors for weight change in our included studies. The baseline characteristics most commonly reported in baseline change weight modelling were chosen for our analysis. Due to the differing measures of effect size, a qualitative approach was used.

### Role of the funding source

The WHO Global HIV, Hepatitis and Sexually Transmitted Infections Programmes, the study sponsor, helped: devise the research question, interpret the data, writing of the manuscript and supporting the decision to submit for publication. Otherwise, the study sponsor had no role in data collection and data analysis. All authors had full access to all the data in the study and take responsibility for the integrity of the data, the accuracy of the data analysis, and the final decision to submit for publication.

## Results

### Evidence base

**Figure 2:**
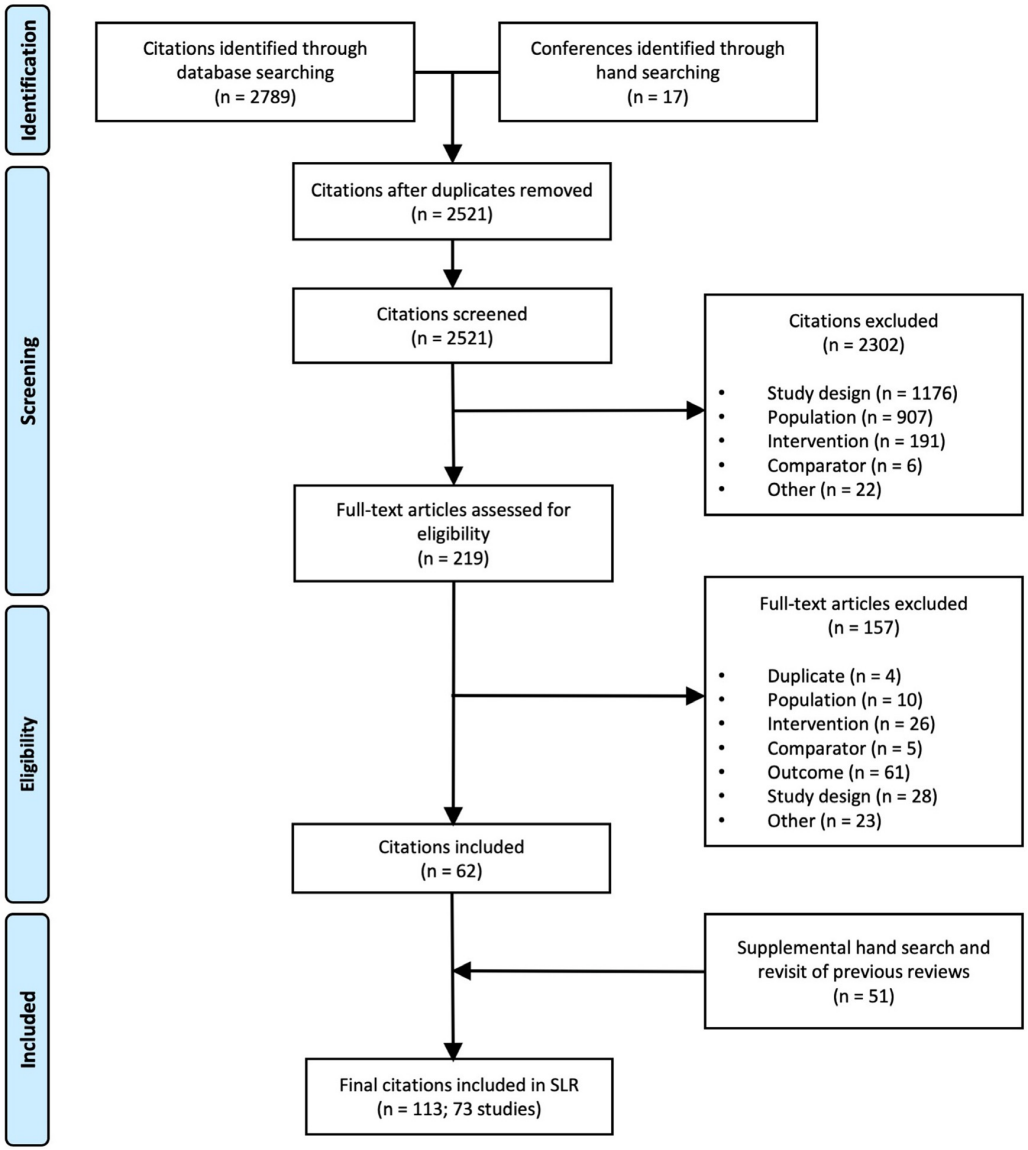
Flow diagram for the systematic literature review.

From the 2521 citations identified through our search strategies, a total of 113 references describing 73 unique studies were included in this analysis. Figure 2 displays the selection process and Figure 1 shows that both networks were well-connected. As our search included terms for outcomes, a larger than normal number of studies were added through supplemental searches due to the inclusion of uncaptured primary publications that reported more extensive study and patient characteristics. Most studies (50/73) were RCTs. In total, the evidence base included 57,124 patients. Not all studies reported weight change. The total number of studies was inflated by the inclusion of hyperglycaemia and of studies reporting prognostic factors. Further study and demographic characteristics are presented in *Web Appendix*.

Overall, study quality for both RCTs and observational studies were generally high with a low risk of bias. Many RCTs were open label, however, the risk of patients switching to treatments other than the one assigned was deemed to be low. For observational studies, several cohort studies did not state the follow-up rate, reducing the assessment score. The full risk of bias assessment can be found in the *Web Appendix*.

### Weight

**Figure 3:**
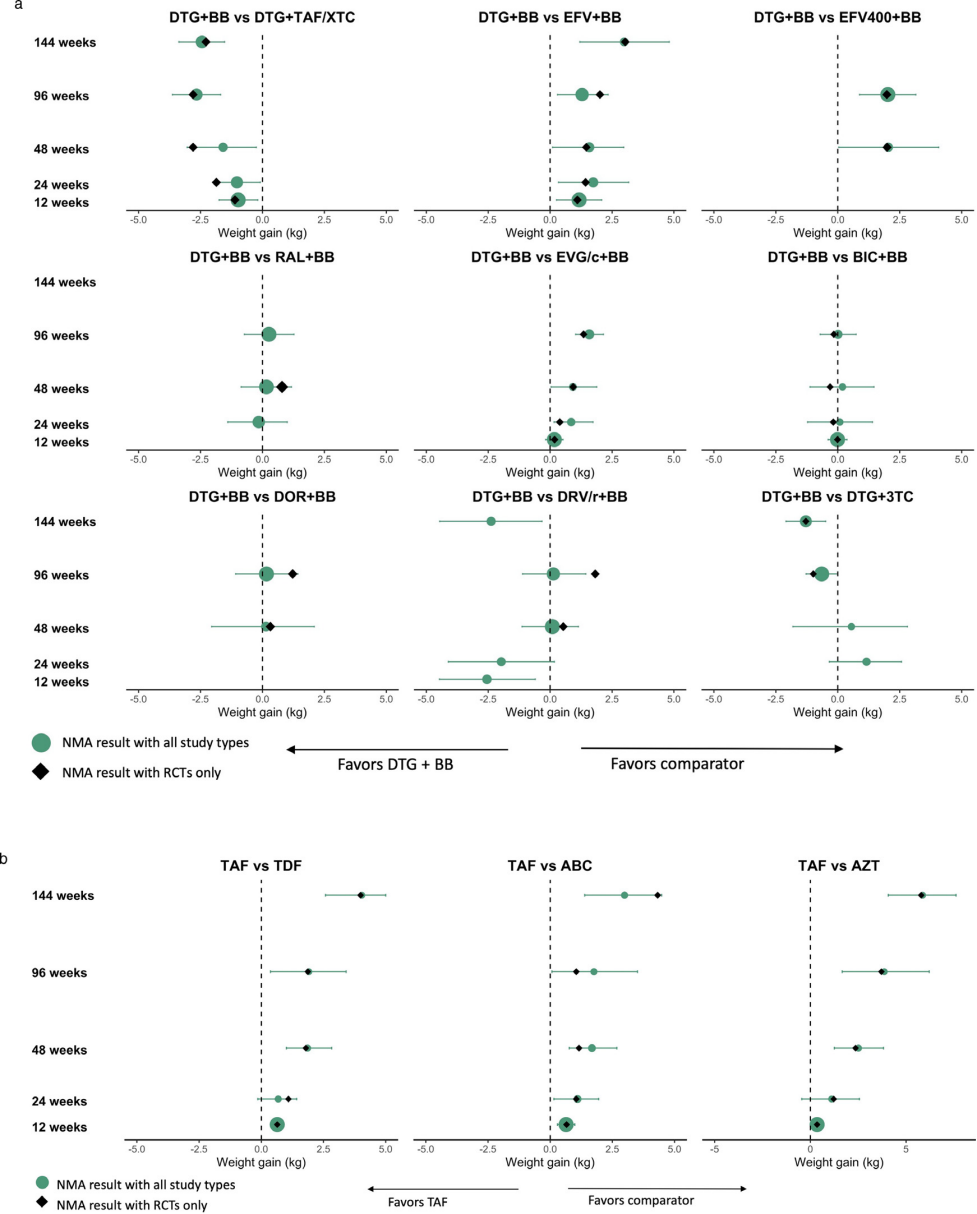
Forest plots for change in weight over time comparing (a) DTG to integrase inhibitors, EFV and other regimens and (b) TAF to other NRTIs according to a series of network meta-analyses. BIC: bictegravir; BB: Back bone (mixed); DTG: dolutegravir; EFV: efavirenz; EVG/c: elvitegravir/cobicistat; RAL: raltegravir; DOR: Doravirine; DRV/r: Ritonavir-boosted darunavir; 3TC: Lamivudine; TAF: tenofovir alafenamide; TDF: Tenofovir disoproxil fumarate; ABC: Abacavir; AZT: Zidovudine; RCT: Randomized controlled trials; NMA: Network meta-analysis. Points represent estimated relative treatment effects for body weight gain using network meta-analyses based on the full evidence base (green circle) and on RCTs only (black diamond). The horizontal lines represent 95% credible intervals for the estimates using the full evidence base. Credible intervals not crossing the dashed vertical line represent a statistically significant difference between the two treatments.

The weight gain analysis focused on the time points that were best reported upon: 12, 24, 48, 96, and 144 weeks. DTG+TAF/XTC had significantly higher weight gains compared to DTG with other NRTI backbones (Figure 3a). Although the addition of observational studies in the analysis reduced the magnitude of the estimate at 24 and 48 weeks, the mean differences remained statistically significant. At 144 weeks, the NMA demonstrated inconsistency and led to a lower relative weight gain estimate compared to the direct evidence (2·44 kg vs. 3·56 kg). DTG also led to higher weight increases relative to NNRTIs and EVG/c, but its body weight gains were comparable to RAL and BIC. The comparison to EFV is of particular interest as body weight gain appears to be stable from weeks 24 to 96, but increases from week 96 to 144. Of note, the body weight gain for EVG/c were non-differentiable to NNRTIs. Differences with PI/r tended not to be statistically significant. For the comparison between DTG and DRV/r, there was a large discrepancy between RCT and observational evidence and results informed only be observational studies should be interpreted with care. Table 1 provides the estimates for the analyses including RCT evidence only.

**Table 1:** Mean differences in change in weight over time comparing (A) DTG to integrase inhibitors and EFV and (B) TAF to other NRTIs, according to a series of network meta-analyses.

	12 weeks	24 weeks	48 weeks	96 weeks	144 weeks
**DTG vs. Other ARVs (Mean differences and 95% credible intervals)**					
DTG+BB vs. DTG+TAF	**−1.11** **kg** **(−1**·**98, −0**·**19)**	**−1·86** **kg** **(−3·08, –0·61)**	**−2·79** **kg** **(−4·42, −1·13)**	**−2·80** **(−3·94, −1·66)**	**−2·28** **(−3·35, −1·23)**
DTG+BB vs. EFV+BB	**1·10** **kg** **(0·17, 2·03)**	**1·43** **kg** **(0·17, 2·67)**	**1·53** **kg** **(0·20, 2·79)**	**2·01** **(0·90, 3·13)**	**3·03** **(1·23, 4·86)**
DTG+BB vs. EFV400+BB	–	–	**2·00** **kg** **(1·18, 2·83)**	**1·98** **(0·89, 3·14)**	–
DTG+BB vs. RAL+BB	–	–	0·81 kg (−2·43, 4·02)	–	–
DTG+BB vs. EVG/*c*+BB	0·18 kg (−0·18, 0·53)	0·39 kg (−0·02, 0·82)	**0·92** **kg** **(0·42, 1·44)**	**1·35** **(0·75, 1·99)**	–
DTG+BB vs. BIC+BB	−0·01 kg (−0·40, 0·38)	−0·18 kg (−0.66, 0.33)	−0·32 kg (−0·93, 0·32)	−0·16 (−0·93, 0·62)	–
DTG+BB vs. DOR+BB	–	–	0·39 kg (−0·91, 1·64)	1·22 (−0·19, 2·62)	–
DTG+BB vs. RPV+BB	−0·05 kg (−1·07, 0·95)	**2·68** **kg** **(1.29, 4·02)**	**3·01** **kg** **(1·54, 4·45)**	**3·31** **(2·00, 4·72)**	–
DTG+BB vs. ATV/*r*+BB	−0·03 kg (−0·61, 0·57)	0·52 kg (−0·30, 1·30)	**1·38** **kg** **(0·37, 2·39)**	0·04 (−2·75, 2·79)	–
DTG+BB vs. DRV/*r*+BB	–	–	0·64 kg (−0·53, 1·78)	**1·82** **(0·19, 3·46)**	–
DTG+BB vs. LPV/*r*+BB	–	**2·50** **kg** **(1·15, 3·89)**	**2·79** **kg** **(1·39, 4·24)**	**3·02** **(1·20, 4·81)**	–
DTG+BB vs. DTG+3TC	–	–	–	**−0·99** **(−1·64, −0·33)**	**−1·29** **(−2·12, −0·51)**
**TAF to other NRTIs (Mean differences and 95% credible intervals)**					
TAF vs. TDF	**0·64** **(0·46, 0·82)**	**1·09** **(0·86, 1·32)**	**1·78** **(1·47, 2·08)**	**1·81** **(1·51, 2·11)**	**3·99** **(2·26, 5·74)**
TAF vs. ABC	**0·66** **(0·30, 1·01)**	**1·05** **(0·59, 1·53)**	**1·15** **(0·59, 1·71)**	**0·88** **(0·27, 1·49)**	**4·32** **(0·53, 8·12)**
TAF vs. AZT	**0·35** **(0·08, 0·60)**	**1·22** **(0·66, 1·78)**	**2·33** **(1·78, 2·88)**	**3·64** **(2·93, 4·36)**	**5·79** **(3·78, 7·82)**

Legend: Bolded values are statistically significant at the 0.05 level. BIC: bictegravir; BB: Back bone (mixed); DTG: dolutegravir; EFV: efavirenz; EVG/c: elvitegravir/cobicistat; RAL: raltegravir; DOR: Doravirine; DRV/r: Ritonavir-boosted darunavir; ATV/r: Ritonavir-boosted atazanavir; LPV/r: Ritonavir-boosted lopinavir; 3TC: Lamivudine; TAF: tenofovir alafenamide; TDF: Tenofovir disoproxil fumarate; ABC: Abacavir; AZT: Zidovudine.

In the TAF analysis, the weight gain were more consistent. TAF showed higher weight gain compared to TDF, ABC, and AZT. As shown in Figure 3b, the relative body weight gain with TAF versus TDF and AZT increased consistently, reaching differences of 4 kg and 5 kg at 144 weeks, respectively (Table 1). This led to TAF being ranked last in all of the analyses it was included in (Web Appendix). The other NRTIs were comparable to one another; however, over longer periods of time both TDF and AZT led to lower weight gain than ABC. Compared to DTG, TAF had higher estimates of relative increases in weight gain.

### BMI and waist circumference

For BMI at 48 weeks, the DTG-based regimens were associated with a statistically significantly higher BMI increases to other INSTIs, with the exception of BIC (mean difference [MD]: −1·47; 95% credible interval [CrI]: −3·36, 0·05). The significant BMI increases ranged from ATV/r (MD: 0·47; 95% CrI: 0·09, 0·83) to EFV (MD: 1·00; 95% CrI: 0·27, 1·68). One notable difference from the body weight analysis was the DTG and RAL comparison. In the body weight analysis, the DTG and RAL comparison showed negligible differences, while the BMI analysis showed DTG leading to larger BMI changes than RAL (MD: 0·13; 95% CrI: 0·06, 0·19). That said, this BMI comparison was entirely informed by observational studies.

Regarding waist circumference at 24 weeks, significance was only seen in the DTG and LPV/r comparison (MD: 1·91; 95% CrI: 0·22, 3·53). At 48 weeks, differences were statistically distinguishable for DTG relative to RAL, EVG/c and to all the PI/r. There was no evidence supporting differences in BMI or waist circumference for TAF. Full results for both these outcomes are presented in the *Web Appendix*.

### Metabolic outcomes

Among these outcomes, only hyperglycaemia was well-reported enough for analysis. The trials reporting diabetes as adverse events never reported more than one case per arm, making an NMA unfeasible. For hyperglycaemia of Grade 2 or higher, the network contained 343 events. The OR relative to EFV was 2·40 (95% CrI: 0·01, 109·34) and relative to ATV/r was 1·31 (95% Cr: 0·04, 13·43). For hyperglycaemia above Grade 3, there were only 43 serious adverse events across a network of 16 studies, creating unreliable estimates with very wide intervals. There were too few events reported in the TAF network to draw meaningful conclusions.

### Prognostic factors

**Figure 4:**
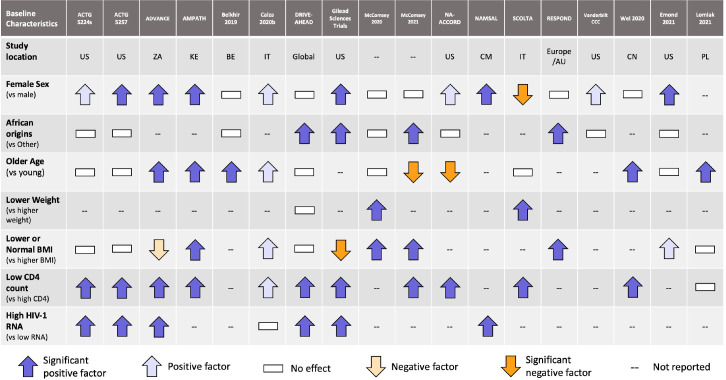
Overview of the prognostic factors analysis for weight change while on antiretroviral therapy. US: United States; ZA: South Africa; KE: Kenya; BE: Belgium; IT: Italy; CM: Cameroon; AU: Australia; CN: China; PL: Poland.

Figure 4 summarizes the results of the identified prognostic factors. Both low CD4 cell count and high HIV RNA at baseline were consistently associated with higher weight gain. CD4 cell count was well reported and all but one study found this to be a significant prognostic factor. The study by Wei et al. (2020)^45^ suggests that increased weight gain is greater in very low CD4 cell counts and that the relationship is only partially linear. This suggests that CD4 cell count may be associated with a return to healthy weight.^46^ Female sex and African origins were also often highlighted as prognostic factors. The analysis conducted on the seven trials by Sax et al. suggests that the effect of sex on weight is modified by ethnicity.^47^ African origin women were more likely to gain more weight compared to African origin men and women of other ethnicities. This relationship is highlighted by studies conducted in Asian, European and American settings, where body weight gain appears to have no association to female sex. Age, baseline BMI, and baseline weight were either inconsistently or sparsely reported, limiting any meaningful conclusions to be drawn.

## Discussion

Our study found evidence that DTG-based regimens lead to larger average weight gains than many other ART regimens and TAF leads to larger average weight gains than all other non-XTC NRTIs. While all ART regimens were observed to lead to average weight gains, ART regimens containing INSTIs were more likely to lead to larger mean body weight gain than other classes. Despite clear class effects, not all treatments were interchangeable within classes. Among INSTIs, EVG/c led to demonstrably lower weight gains than others. Among NRTIs, the trend was clearest: TAF stands out as leading to demonstrably larger mean weight gains than all other NRTIs. Indeed, while there tended to be moderate certainty of evidence for DTG compared to key comparators, with some instances of low certainty, the comparison between TAF and TDF was of moderate certainty that TAF leads to larger mean weight gains. Our study also found a rich evidence base of prognostic factors analyses that help shed light on the weight gain risks among people living with HIV that are on ART.

The signal of potential larger average weight gains with DTG was first raised by the ADVANCE trial.^13^ Since then, more evidence of weight gain among ART users has been published. Given the chronic nature of the disease, it is imperative to monitor patients for important, unhealthy weight gains as these issues can lead to increased risk to non-communicable diseases,^29^ particularly as the population ages.^48^ The increased evidence base has helped confirm that there are larger weight gains with DTG and other INSTIs relative to other treatments. These results are in agreement with a review of literature by Eckard and McComsey concluded INSTIs cause more weight gain compared to non-INSTIs.^24^ Importantly, our study also helped establish the weight gain associated with TAF relative to other NRTIs. However, it is important to recognize that although our analyses looked at mean weight gain, the observed weight gains were not normally distributed among patients. Only a specific proportion of patients experienced weight gains. Moreover, weight gains can be separated into two categories: (1) gain in weight back to normal after an episode of sickness; and (2) excessive weight gain leading overweight or obesity. The manner by which the results of studies are reported did not allow for these categories to be distinguished in the analyses.

This study explored the prognostic factors of weight gain. While meaningful conclusions were made, more analyses are needed to distinguish the factors associated with a “return to normal” weight or excessive weight gains from other sources. Our review found that low CD4 cell count and high HIV RNA are both consistently associated with higher body weight gain; that African origin is associated with larger weight gain as well; and that female sex is an effect-modifier to African origin with respect to weight gain. However, both low CD4 cell count and high HIV RNA levels are markers of a more advanced HIV disease. Treatment of HIV via initiation of ART could create these “return to normal” gains in body weight. This increase in body weight is suggested to be from the reversal of HIV-related inflammation and restoration of normal catabolism; although the exact reasons are still not certain.^19^ It is necessary to further understand which factors govern each type of body weight gain. While good progress has been made on prognostic factors of weight gains, analyses that distinguish patients within different groups could be more informative for the future. Our analysis also suggests that the effect of TAF and DTG might be additive given the results of TAF vs TDF and of DTG+TAF/XTC vs DTG+BB (only the 144-week results are differentiable from one another; i.e., not lending themselves to an additive effect). Determining this additive effect should be a continued avenue of research.

Key metabolic outcomes were explored in this study. There were no statistically significant results of increased risk of hyperglycemia or diabetes mellitus among patients using DTG; however, estimates suggested higher risk. We understand that the use of ARTs will increase the risk of diabetes, but more studies will be needed to further explore the risk created by specific ART regimens.^29^ Given that weight gains may lead to these outcomes but our results are not normally distributed, it might be more useful to explore the risks of these metabolic outcomes among those that have experienced important weight gain.

Our study has numerous strengths and limitations. First, the expansive SLR led to a large evidence base that comprehensively identified the current state of research. Second, by reviewing all the analyses of the prognostic factors, we were able to draw stronger conclusions than in the individual analyses. With respect to limitations, firstly, the inclusion of observational studies does introduce a risk of bias. Analyses without observational studies were conducted, and their impact appears to be negligible. Alterative, more sophisticated methods could have been used to account for multiple study designs, but these methods find estimates between the models with and without the observational studies. Therefore, little was lost in not employing these other methods. Secondly, metabolic outcomes were limited by a very low number of events, reducing the statistical power. Thirdly, despite a much richer evidence base than in 2019, the number of studies informing individual comparisons were low for many of the networks, rendering both random-effects and meta-regression difficult and at times infeasible. To this end, an analysis mixing both aggregate data and individual patient data could lead to improved results. Importantly, most of the trials from the DTG development program (e.g., SINGLE, FLAMINGO, DAWNING, etc.) did not report weight changes. Having these data would have avoided this limitation. Fourthly, the systematic review did not include long-acting regimens as they are not yet available in LMIC settings, but understanding how these compare to current evidence base is of interest. Finally, we did collect data on visceral fat and total fat distribution outcomes, but there was simply not enough data on DTG and TAF to warrant analysis.

In summary, there is moderate certainty that DTG-based regimens lead to larger body weight gains than EFV, EFV_400_ and EVG/c. There is also moderate certainty of evidence that DTG combined with TAF containing backbones leads to larger body weight gain than DTG combined with other NRTI backbones and that weight gain in TAF-based regimens relative to TDF-based regimens. With respect to prognostic factors, both low CD4 cell counts and high HIV RNA levels are highly indicative of higher body weight gain, while the effects of sex on weight gain appear to be ethnicity dependent. Further research is needed to better understand long-term body weight gains and how they relate to other metabolic outcomes.

## Declaration of interests

Steve Kanters, Eve Limbrick-Oldfield, and Kenneth Zhang are employees at RainCity Analytics. All other authors have nothing to declare.

## Appendix Supplementary materials

Image, application 1
