# Supplementary material for: Evidence synthesis evaluating body weight gain among people treating HIV with antiretroviral therapy - a systematic literature review and network meta-analysis
Source: eClinicalMedicine. 2022 May 12;48:101412. doi: 10.1016/j.eclinm.2022.101412 (PMC9112095; doi:10.1016/j.eclinm.2022.101412)
Supplement: Supplementary file 1 [file mmc1.docx]

Web Appendix: Supplementary methods and results
